# Powerful gene network enrichment analysis and its application to severe COVID-19 gene network

**DOI:** 10.1093/bib/bbaf647

**Published:** 2025-12-05

**Authors:** Heewon Park, Seiya Imoto, Satoru Miyano

**Affiliations:** School of Mathematics, Statistics and Data Science, Sungshin Women’s University, 2, 34 da-gil, Bomun-ro, Seongbuk-gu, Seoul, 02844, Republic of Korea; M&D Data Science Center, Institute of Integrated Research, Institute of Science Tokyo, 1-5-45 Yushima, Bunkyo-ku, Tokyo 113-8510, Japan; Human Genome Center, Institute of Medical Science, University of Tokyo, 4-6-1 Shirokanedai, Minato-ku, Tokyo, 108-8639, Japan; Data Science Center, Sungshin Women’s University, 2 Bomun-ro 34 da-gil, Seongbuk District, Seoul, Republic of Korea; Human Genome Center, Institute of Medical Science, University of Tokyo, 4-6-1 Shirokanedai, Minato-ku, Tokyo, 108-8639, Japan; M&D Data Science Center, Institute of Integrated Research, Institute of Science Tokyo, 1-5-45 Yushima, Bunkyo-ku, Tokyo 113-8510, Japan; Human Genome Center, Institute of Medical Science, University of Tokyo, 4-6-1 Shirokanedai, Minato-ku, Tokyo, 108-8639, Japan

**Keywords:** gene network, functional pathway analysis, COVID-19, viral infection disease

## Abstract

Understanding complex disease mechanisms requires research methods beyond individual gene analysis to capture the coordinated behavior of genes within regulatory networks. Traditional gene set enrichment approaches such as over-representation analysis and gene set enrichment analysis focus primarily on gene lists and often overlook the intricate network structures that control cellular processes. Although a gene network enrichment analysis strategy (GbNEA) has been proposed, this method assesses enrichment significance via phenotype permutation and the Kolmogorov–Smirnov test, which lowers statistical power and increases the computational burden due to repeated gene network re-estimation. To overcome these limitations, we developed a novel approach, powerful gene network enrichment analysis (PGNEA), which characterizes gene networks by integrating gene expression, regulatory effects, and hubness. PGNEA evaluates the enrichment of phenotype-specific gene networks by quantifying differences in gene activity patterns and assesses statistical significance by evaluating permutation of gene activity rather than phenotype permutation. This approach exhibits significantly enhanced computational efficiency and statistical sensitivity. We demonstrated the advantages of PGNEA through Monte Carlo simulations and applied it to whole-blood RNA-seq data obtained from the Japan COVID-19 Task Force. PGNEA successfully identified viral infection-related pathways enriched in severe COVID-19 gene networks, including those linked to “COVID-19,” “HIV-1 infection,” “Hepatitis B,” “Influenza A,” “Measles,” and “Kaposi sarcoma-associated herpesvirus infection.” Notably, key molecular markers such as PIK3, NF-B family members, FOXA, JUN, and CXCL8 were identified, with strong and consistent molecular interplays between CXCL8 and NFKBIA. These findings underscore the potential of PGNEA as an efficient tool for identifying biologically meaningful pathways and network-level mechanisms associated with various phenotypes, including severe viral infections.

## Introduction

Understanding the complex molecular mechanisms underlying human diseases requires not only the identification of individual genes but also the elucidation of their coordinated actions within biological networks. Recent advances in systems biology have highlighted the importance of gene network-based analyses that consider the collective behavior of genes and their interactions. Although numerous computational strategies have been developed for gene network estimation and network-based analysis approaches have garnered considerable attention, few studies have been devoted to the interpretation of large-scale gene networks. Gene networks typically comprise over 10 000 genes with several million edges due to their inherent complexity and high dimensionality. Thus, interpreting gene networks remains a highly challenging task.

Extensive research has been conducted using gene set enrichment analysis (GSEA) to aid in the biological interpretation of predefined gene sets, such as over-representation analysis (ORA) [1] and GSEA [2]. Several network-based gene set enrichment methods have been developed, including NEAT, which evaluates enrichment based on gene associations [3], and GSCE, which assesses the relationships between differentially expressed genes and functional sets using co-expression networks [4]. However, existing studies have primarily focused on individual gene lists and have often overlooked the intricate regulatory relationships and network structures that govern cellular processes. Consequently, these approaches may fail to capture the full spectrum of molecular interactions that drive phenotypic variation.

Gene network enrichment analysis aims to determine whether molecular interplay within a specific network is associated with a phenotype of interest, offering a more comprehensive view of disease mechanisms. Park *et al.* [5] developed gene behavior-based network enrichment analysis (GbNEA), which assesses the significance of the enrichment score based on a permutation framework in which the phenotype is permuted. Permuted gene networks are estimated based on the permuted cell lines within the phenotype, and the significance of the enrichment score was measured by comparing an enrichment score with a set of permuted scores. The permutation phenotype was used to generate a null distribution under the hypothesis that the gene network contains no molecular interplays associated with the phenotype of interest. In contrast, GbNEA employs the Kolmogorov–Smirnov test based on the same permutation scheme to assess whether the association patterns in the query network differ from those in the background network. This results in the loss of statistical power [6]. Furthermore, permuted gene network estimation significantly increases computational complexity.

**Figure 1:**
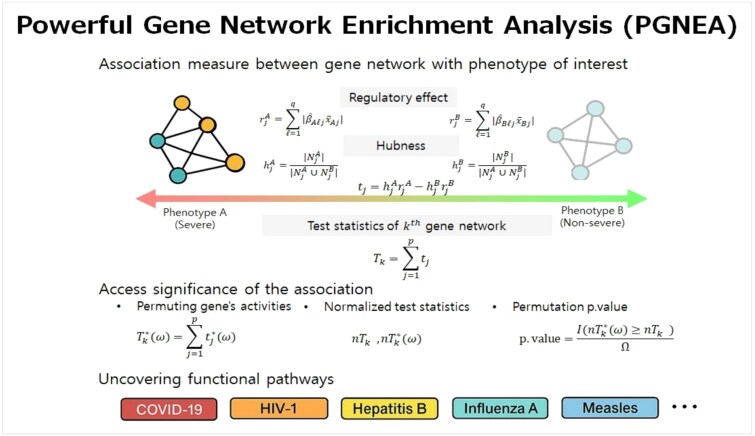
The overall framework of the proposed PGNEA: We first compute gene activities based on regulatory effect and hubness and then evaluate the association between gene networks and the phenotypes of interest, assessing its significance by permuting genes rather than phenotypes within networks and calculating the resulting permuted p-value.

To address these challenges, we propose a novel computational strategy named powerful gene network enrichment analysis (PGNEA), which effectively assesses the association between gene networks and phenotypic traits. Our strategy characterizes gene networks by capturing the coordinated behavior of genes, including expression levels, regulatory effects, and hubness. We evaluated network enrichment by quantifying differences in the activities of genes in a network between phenotypes, allowing for the effective identification of functional pathways enriched in phenotype-specific gene networks. Importantly, statistical significance is assessed by permuting gene activities in a network rather than phenotypes, which eliminates the need to re-estimate gene networks under permutation. This approach results in improved statistical power and computational efficiency. An overview of the proposed PGNEA framework is illustrated in Figure 1.

We demonstrated the effectiveness of the PGNEA in uncovering functional pathways enriched in phenotype-specific gene networks using Monte Carlo simulations. Additionally, we applied PGNEA to whole-blood RNA-seq data provided by the Japan COVID-19 Task Force [7] and identified viral infection-related pathways enriched within severe COVID-19 gene networks. Our analysis revealed significant enrichment of several viral infection pathways, including “Coronavirus disease - COVID-19,” “HIV-1 infection,” “Hepatitis B,” “Influenza A,” “Measles,” and “Kaposi sarcoma-associated herpesvirus (KSHV) infection.” Furthermore, key molecular markers, such as members of the PIK3 and NF-B families, along with FOX, JUN, and CXCL8, were detected. Interestingly, a strong association between CXCL8 and NFKBIA was consistently observed as a shared signature across enriched viral infection pathways associated with severe COVID-19 gene networks. These identified markers were further corroborated by the findings of existing literature. Collectively, our findings suggest that targeting these pathways and identifying the molecular interactions could provide valuable insights into the pathogenesis of severe COVID-19 and other viral infectious diseases.

The remainder of this paper is organized as follows: The Methods section introduces the proposed strategy, PGNEA. The Monte Carlo simulations subsection within the Results section presents the outcomes of simulation studies. Subsequently, the identification results of viral infection disease pathways related to severe COVID-19 gene networks are described. Finally, the Discussion section interprets these findings and provides concluding remarks.

## Powerful gene network enrichment analysis

The objective of gene network enrichment analysis is to test whether molecular interplay is associated with the phenotype of interest. To address this research objective, we considered the following hypothesis:

**Null hypothesis**: *The molecular interplays in a query network show the same pattern of associations with the phenotype compared with the molecular interplays in other networks.*

To test this hypothesis, we examined whether the observed association of molecular interplay with the phenotype of interest was a random sample from the background distribution of all observed associations. Thus, the main objective of the gene network enrichment analysis was to characterize the association between molecular interplay and the phenotypes of interest. To characterize this association, we considered the behavior of the genes within the network as the subject of enrichment analysis.

With indices $i$ and $j$ denoting the $j{\mathrm{th}}$ and $i{\mathrm{th}}$ cells, respectively, $i=1,...,n, j=1,...,p$. We assumed that the phenotype of interest is measured by $\{z_{1},...,z_{n}\}$ with the resulting association measure $t_{j}$ between the interplay of the $j{\mathrm{th}}$ gene and the phenotype of interest. We considered the following matrix to describe the gene network information [6]: 


\begin{align*} \begin{pmatrix} t_{1} & t_{2} &\cdots & t_{p}\\ g_{11} & g_{12} &\cdots& g_{1p}\\ \vdots & \vdots &\ddots& \vdots\\ g_{k1} & g_{k2} &\cdots& g_{kp}\\ \end{pmatrix}\nonumber\end{align*}

where $g_{kj}, k=1,...,K$, and $j=1,...,p$ are indices of the $k{\mathrm{th}}$ networks of interest; i.e. $g_{kj}=1$ if the $k{\mathrm{th}}$ network contains interplays of the $j{\mathrm{th}}$ gene, and $0$ otherwise.

### Gene network estimation

Suppose that $\boldsymbol{X} = (\boldsymbol{x}_{1}, \dots , \boldsymbol{x}_{n})^{T} \in \mathbb{R}^{n \times p}$ is an $n \times p$ data matrix describing the expression levels of $p$ regulator genes in $n$ cell lines. Let $\boldsymbol{y}_{\ell } \in \mathbb{R}^{n}$ denote the expression levels of the $\ell{\mathrm{th}}$ target gene, for $\ell = 1, \dots , q$. We considered a directed gene regulatory network and used the following linear regression model to describe the gene network: 


(1) \begin{gather*} y_{i\ell}=\boldsymbol{\beta}^{T}_{\ell}\boldsymbol{x}_{i}+\epsilon_{i\ell}, \quad \ell=1,...,q, \end{gather*}

where $\boldsymbol{\beta }_{\ell } = (\beta _{\ell 1}, \dots , \beta _{\ell p})^{T} \in \mathbb{R}^{p}$ and $\boldsymbol{\varepsilon }_{\ell } = (\epsilon _{\ell 1}, \dots , \epsilon _{\ell n})^{T} \in \mathbb{R}^{n}$ is a random error vector accounting for residual variation. The edge weight (i.e. $\beta _{\ell j}$) estimation and edge selection were conducted using LASSO [8], 


(2) \begin{gather*} \hat{\boldsymbol{\beta}}_{\ell}=\mathop{\mathrm{arg~min}}_{\boldsymbol{\beta}_{\ell}}\left\{\frac{1}{2}\sum^{n}_{i=1}(y_{i\ell}-\boldsymbol{\beta}^{T}_{\ell}\boldsymbol{x}_{i})^{2}+\lambda\sum_{j=1}^{p}|\beta_{\ell j}|\right\}, \end{gather*}

where $\lambda>0$ is a hyperparameter for controlling the degree of shrinkage of $\boldsymbol{\beta }_{\ell }$. We estimated the gene networks for phenotypes A and B separately using the expression data specific to each phenotype.

### A novel association measure between gene networks and the phenotype of interest

#### Characteristics of gene’s activities in network

1. **Regulatory effects**
  The regulatory effects of the $j{\mathrm{th}}$ regulatory genes in the gene network were estimated as follows [5, 9]:
  (3) \begin{gather*} r^{A}_{j}=\sum_{\ell=1}^{q}|\hat{\beta}_{A\ell j}\bar{x}_{Aj}| \mathrm{and} r^{B}_{j}=\sum_{\ell=1}^{q}|\hat{\beta}_{B\ell j}\bar{x}_{Bj}|, \end{gather*}
  where $\hat{\beta }_{A\ell j} (\hat{\beta }_{B\ell j})$ and $\bar{x}_{Aj} (\bar{x}_{Bj})$ are the estimated edge weights from the $j{\mathrm{th}}$ regulatory gene to the $\ell{\mathrm{th}}$ target gene and the average of expression levels of the $j{\mathrm{th}}$ gene for the phenotype $A (B)$, respectively. The regulatory effects $r^{A}_{j}$ and $r^{B}_{j}$ measure the strength of the effect of the $j{\mathrm{th}}$ gene on the gene networks of phenotypes $A$ and $B$, respectively.
2. **Hubness**: Degree of connectivity
  We considered the following hubness a characteristic of the genes.
  (4) \begin{align*} h^{A}_{j}=\frac{|N^{A}_{j}|}{|N^{A}_{j}\cup N^{B}_{j}|} \mathrm{and} h^{B}_{j}=\frac{|N^{B}_{j}|}{|N^{A}_{j}\cup N^{B}_{j}|},\end{align*}
  where $0\leq h^{A}_{j}, h^{B}_{j}\leq 1$, $N^{A}_{j}$, and $N^{B}_{j}$ are the sets of nodes that are directly connected to the $j{\mathrm{th}}$ gene in the two networks of phenotypes $A$ and $B$, respectively. Large values of $h^{A}_{j} (h^{B}_{j})$ indicate that the $j{\mathrm{th}}$ gene is a hub gene. Due to their centrality, even slight changes in the expression of hub genes often lead to widespread effects across the entire network architecture. Therefore, genes with large values of $h^{A}_{j}$ (or $h^{B}_{j}$) are likely to significantly influence the overall network structure.
3. **Activities of the $j{\mathrm{th}}$ gene in the networks**:
  Additionally, we considered the following statistic to measure activities of genes by combining the regulatory effect and hubness of a gene,
  (5) \begin{align*} h^{A}_{j}r^{A}_{j}=\frac{|N^{A}_{j}|}{|N^{A}_{j}\cup N^{B}_{j}|}\sum_{\ell=1}^{q}|\hat{\beta}_{A\ell j}\bar{x}_{Aj}|,\\ h^{B}_{j}r^{B}_{j}=\frac{|N^{B}_{j}|}{|N^{A}_{j}\cup N^{B}_{j}|}\sum_{\ell=1}^{q}|\hat{\beta}_{B\ell j}\bar{x}_{Bj}|. \nonumber\end{align*}

#### Measurement of the association between gene networks and the phenotype of interest

To capture the differences in gene behavior across networks, we defined the fold-change in gene activity, 


(6) \begin{align*} t_{j}=h^{A}_{j}r^{A}_{j}-h^{B}_{j}r^{B}_{j}.\end{align*}

Then, we propose utilizing the following statistics to test the $k{\mathrm{th}}$ gene network: 


(7) \begin{align*} T_{k}=\frac{1}{m_{k}}\sum_{j=1}^{p}g_{kj}t_{j},\end{align*}

where $m_{k}=\sum _{j=1}^{p}g_{kj}$ denotes the number of genes in the $k{\mathrm{th}}$ network. A large $T_{k}$ value indicates that the $k{\mathrm{th}}$ gene network has a coordinated association with the phenotype of interest.

#### Assessment of the significance of the enrichment

We assessed the significance of the enrichment of a query network by comparing it with a set of permuted test statistics. The null distribution of $T_{k}$ was generated by permuting $\{t_{1},...,t_{p}\}$. In other words, under the null hypothesis, the null distribution of $(T_{1},...,T_{K})$ can be approximated using the empirical distribution of $(T^{*}_{1}(\omega ),...,T^{*}_{K}(\omega ))$, where 


(8) \begin{align*} T^{*}_{k}(\omega)=\frac{1}{m_{k}}\sum_{j=1}^{p}g_{kj}t^{*}_{j}(\omega), \quad k=1,...,K, \omega=1,...\Omega,\end{align*}

where $\Omega $ is the number of permutations and $\{t^{*}_{1}(\omega ),...,t^{*}_{p}(\omega )\}$ is the permuted $\{t_{1},...,t_{p}\}$.

We normalized $T_{k}$ and $T^{*}_{k}(\omega )$, thereby rescaling the positive and negative scores by dividing them by the mean of $T^{*}_{k}(\omega )$ to compute the normalized enrichment scores $nT^{*}_{k}(\omega )$ and $nT_{k}$ in line with [2].

Next, we computed the permutation *p*-value as follows: 


(9) \begin{align*} p-\mathrm{value}=\frac{\sum_{\omega=1}^{\Omega}I(nT^{*}_{k}(\omega) \geq nT_{k})}{\Omega},\end{align*}

where $I(\cdot )$ is the indicator function. We consider the query gene network is statistically significant when the *p*-value was less than the significance level $\alpha $. The Benjamini–Hochberg procedure was applied to account for multiple tests, and the resulting *p*-values were used to control the false discovery rate (FDR) [10]. Our strategy adopts a distinct permutation framework, i.e. permuting $\{t_{1},...,t_{p}\}$ unlike the existing GbNEA, which permutes the phenotype $\{z_{1},...,z_{n}\}$ [6]. This approach allows us to overcome the limitations of GbNEA while enhancing both statistical power and computational efficiency, as described below.

- **Statistical power**
  The permuting phenotypes of GbNEA (i.e. $\{z_{1}\cdots z_{n}\}$) generates the null distribution of the test statistic under the hypothesis “the gene network does not contain any molecular interplays associated with phenotype of interest.” On the other hand, GbNEA test “the molecular interplays in a query network show the same pattern of association with the phenotype compared with the molecular interplays in other networks” by using Kolmogorov–Smirnov test. That is, there is an incongruity between the null distribution of the test statistic and the testing framework. This mis-specified null distribution results in a reduction of the statistical test’s power. Our strategy correctly rectifies the previously mis-specified null distribution of the test statistic. That is, PGNEA permutes the set of gene’s activities (i.e. $\{t_{1},\cdots , t_{p}\}$) across networks, thereby generating a valid null distribution of the test statistic $T_{k}$ corresponding to the hypothesis: “The molecular interactions within the query network display a pattern of phenotype associations consistent with those observed in other networks.” By permuting the gene’s activities rather than the phenotypes, we generate an appropriate null distribution of the test statistic for our null hypothesis, and thus we can enhance statistical power of the enrichment analysis.
- **Computational efficiency**
  GbNEA involves estimating networks for each phenotype and subsequently computing a test statistic to assess the difference in gene behavior. The null distribution of the test statistics was generated by permuting phenotypes. That is, the gene networks of two phenotypes were estimated based on the permuted cell lines, and thus permuted networks should be estimated $\Omega $ times. Inferring gene networks demands considerable computational resources by itself; on top of this, performing the estimation for $\Omega $ permutation samples significantly increases the computational burden. This is a critical limitation of the GbNEA. On the other hand, our strategy permutes gene’s activities in the pre-estimated gene network rather than phenotypes, thereby eliminating the need to re-estimate gene networks under permutation. Consequently, this approach achieves greater computational efficiency.

## Results

### Simulation studies

Simulations were conducted to assess the performance of the proposed PGNEA. We evaluated our strategy by comparing it with GbNEA based on the publicly available DepMap (https://depmap.org/portal/) and Kyoto Encyclopedia of Genes and Genomes (KEGG pathways (https://www.genome.jp/kegg/pathway.htm) database used in the initial study on GbNEA. We considered cancer pathways (i.e. *cancer: specific types*) as benchmark scenarios for GbNEA.

The gene network was estimated using the CCLE expression dataset from the DepMap database, which comprises 19 221 genes and 1406 cells. Table 1 lists cancer pathways from the KEGG pathway database and the number of cell lines for each cancer type in the CCLE expression dataset.

**Table 1:** Cancer-related pathways in the KEGG database and numbers of cell lines in the CCLE expression data

Entry	Name	No. Genes	No. Cells
hsa05210	Colorectal cancer	87	85
hsa05212	Pancreatic cancer	77	60
hsa05226	Gastric cancer	150	48
hsa05223	NSCL cancer	73	156
hsa05222	SCL cancer	93	77

For the gene network enriched in nonsmall lung cancer (NSCL), we considered the 73 NSCL-related genes defined in the KEGG database and randomly selected $p-73$ genes from those with the 2000 highest variance in expression. Then, we estimated two gene networks for the two phenotypes (i.e. NSCL and normal cells) based on the expression levels of the extracted $p$ genes in 156 NSCL and 103 noncancerous cell lines (i.e. NSCL cancer networks). Subsequently, we computed the statistics $t_{j}, j=1,...,p$ for the genes in the NSCL networks and the test statistic $T_{\mathrm{NSCL}}$. For the permutation, we also estimated two networks containing 2000 genes with the highest variance in expression based on 100 randomly selected cell lines (i.e. random networks) and computed $t^{*}_{j}(\omega ), j=1,...,2000$. The permuted statistic $T^{*}_{\mathrm{NSCL}}(\omega )$ was computed using permuted gene activities (i.e. $t_{j}$) from both NSCL and random networks. Finally, we compute the permutation *p*-value based on the normalized test statistics $nT_{\mathrm{NSCL}}$ and $nT^{*}_{\mathrm{NSCL}}(\omega )$. When the estimated NSCL gene networks showed significant enrichment ( $P<.05$), this was regarded as a true positive in the gene network enrichment analysis. For the true-negative scenario, we estimated a false NSCL gene network based on randomly selected 5% of 73 NSCL genes (i.e. four genes) and randomly selected $p-4$ genes, using the expression levels of 156 NSCL and 103 noncancerous cell lines. We classified the results as false positives when the NSCL pathway was significantly enriched in the false network. Similar analyses were performed for other cancer-related pathways including colorectal, pancreatic, gastric, and small cell lung (SCL) cancers.

The effectiveness of gene network enrichment was benchmarked against GbNEA and other strategies for GSEA, including ORA, GSEAn, and GSEAc, in which genes in a network were considered as gene sets [11]. All the comparative methods were performed using the *gsean* package in R [11].

**Figure 2:**
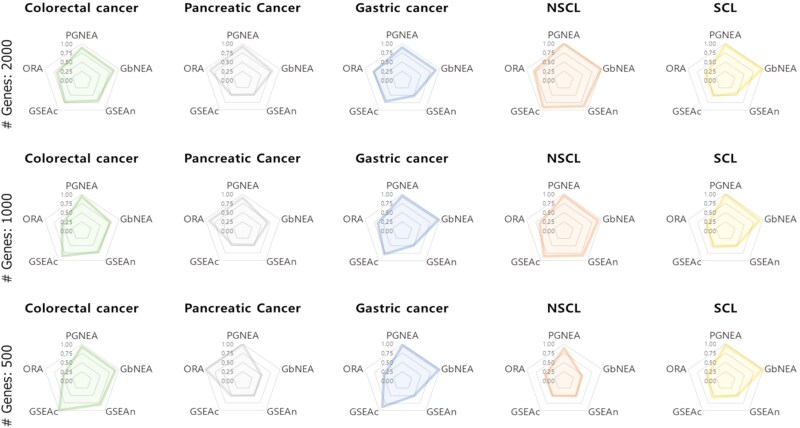
Accuracy of gene network enriched pathway identification. (ORA, Over-representation analysis; GbNEA, Gene behavior-based network enrichment analysis; GSEAn, Gene set enrichment analysis with network structure; GSEAc, Gene set enrichment analysis with centrality measure).

Figure 2 shows the accuracy of the GSEA with different numbers of genes $p=500, 1000, 2000$. As shown in Fig. 2, the gene network-based strategies, i.e. PGNEA and GbNEA, outperformed the common methods regarding uncovering enrichment pathways in the query gene network, especially the proposed PGNEA, which showed outstanding overall performance.

We evaluated the methods based on the following metrics: accuracy, precision, recall, F1 score, true positive rate (TPR) and true negative rate (TNR). 


\begin{align*} &\mathrm{TPR}=\frac{\mathrm{TP}}{\mathrm{Actual\ positives}},\quad \mathrm{TNR}=\frac{\mathrm{TN}}{\mathrm{Actual\ negatives}},\\ &\mathrm{Accuracy}=\frac{\mathrm{TP}+\mathrm{TN}}{\mathrm{TP}+\mathrm{FP}+\mathrm{FN}+\mathrm{TN}},\quad \mathrm{Recall}=\frac{\mathrm{TP}}{\mathrm{TP}+\mathrm{FN}},\\ &\mathrm{Precision}=\frac{\mathrm{TP}}{\mathrm{TP}+\mathrm{FP}},\quad \mathrm{F1\ score}=\frac{2\times \mathrm{Precision}\times \mathrm{Recall}}{\mathrm{Precision}+ \mathrm{Recall}}. \end{align*}

Table 2 presents the results of gene network enrichment pathway analysis based on various evaluation metrics (i.e. accuracy, precision, recall, F1 score), where “–” denotes cases in which precision could not be calculated due to the absence of identified pathways. A complete presentation of the evaluation results can be found in the supplementary file.

**Table 2:** Gene network enrichment analysis results, with bold numbers indicating the best-performing method

		No. Genes: 500				No. Genes: 1000				No. Genes: 2000			
		Accuracy	Precision	Recall	F1.score	Accuracy	Precision	Recall	F1.score	Accuracy	Precision	Recall	F1.score
Coloreactal	PGNEA	**0.89**	0.82	1.00	0.90	**0.95**	0.91	1.00	0.95	0.94	0.89	1.00	0.94
	GbNEA	0.86	0.95	0.76	0.84	0.78	1.00	0.56	0.72	0.91	1.00	0.82	0.90
	GSEAn	0.69	0.88	0.44	0.59	0.71	0.84	0.52	0.64	0.81	1.00	0.62	0.77
	GSEAc	0.73	0.87	0.54	0.67	0.86	0.91	0.80	0.85	**1.00**	1.00	1.00	1.00
	ORA	0.67	0.60	1.00	0.75	0.54	0.52	1.00	0.68	0.50	0.50	1.00	0.67
Pancreatic	PGNEA	**0.93**	0.88	1.00	0.93	**0.91**	0.85	1.00	0.92	0.99	0.98	1.00	0.99
	GbNEA	0.80	0.88	0.70	0.78	0.68	0.95	0.38	0.54	0.51	0.67	0.04	0.08
	GSEAn	0.49	0.00	0.00	0.00	0.48	0.00	0.00	0.00	0.50	–	0.00	0.00
	GSEAc	0.50	0.50	0.02	0.04	0.46	0.00	0.00	0.00	0.51	1.00	0.02	0.04
	ORA	0.88	0.81	1.00	0.89	0.90	0.83	1.00	0.91	**1.00**	1.00	1.00	1.00
Gastric	PGNEA	**0.90**	0.83	1.00	0.91	**0.95**	0.91	1.00	0.95	0.96	0.93	1.00	0.96
	GbNEA	**0.90**	0.90	0.90	0.90	0.97	0.94	1.00	0.97	**0.97**	0.94	1.00	0.97
	GSEAn	0.52	1.00	0.04	0.08	0.49	0.00	0.00	0.00	0.50	–	0.00	0.00
	GSEAc	0.71	1.00	0.42	0.59	0.78	0.97	0.58	0.73	0.88	1.00	0.76	0.86
	ORA	0.76	0.68	1.00	0.81	0.66	0.60	1.00	0.75	0.50	0.50	1.00	0.67
NSCL	PGNEA	**0.99**	0.98	1.00	0.99	**0.96**	0.93	1.00	0.96	**0.89**	0.82	1.00	0.90
	GbNEA	**0.99**	0.98	1.00	0.99	0.92	0.90	0.94	0.92	0.47	0.94	0.50	0.65
	GSEAn	0.87	0.85	0.90	0.87	0.83	0.75	0.98	0.85	0.52	0.51	1.00	0.68
	GSEAc	0.90	0.84	0.98	0.91	0.86	0.78	1.00	0.88	0.50	0.50	1.00	0.67
	ORA	0.80	0.71	1.00	0.83	0.65	0.59	1.00	0.74	0.50	0.50	1.00	0.67
SCL	PGNEA	0.97	0.94	1.00	0.97	**0.99**	0.98	1.00	0.99	0.97	0.94	1.00	0.97
	GbNEA	**0.99**	1.00	0.98	0.99	0.91	0.86	0.98	0.92	**0.98**	0.96	1.00	0.98
	GSEAn	0.45	0.00	0.00	0.00	0.50	–	0.00	0.00	0.50	–	0.00	0.00
	GSEAc	0.52	0.58	0.14	0.23	0.54	1.00	0.08	0.15	0.56	1.00	0.12	0.21
	ORA	0.53	0.52	1.00	0.68	0.50	0.50	1.00	0.67	0.50	0.50	1.00	0.67
Average	PGNEA	**0.94**	0.89	1.00	0.94	**0.95**	0.91	1.00	0.95	**0.95**	0.91	1.00	0.95
	GbNEA	0.91	0.94	0.87	0.90	0.85	0.93	0.77	0.81	0.77	0.90	0.67	0.72
	GSEAn	0.60	0.55	0.28	0.31	0.60	0.40	0.30	0.30	0.57	0.76	0.32	0.29
	GSEAc	0.67	0.76	0.42	0.49	0.70	0.73	0.49	0.52	0.69	0.90	0.58	0.56
	ORA	0.73	0.66	1.00	0.79	0.65	0.61	1.00	0.75	0.60	0.60	1.00	0.73

As shown in Table 2, PGNEA and GbNEA, both gene network-based strategies achieved relatively better performance than conventional gene-based approaches. The proposed strategy exhibited the best overall results. GSEAn and GSEAc performed reasonably well in terms of TN, yet their effectiveness in identifying true positives was limited. In contrast, ORA demonstrated weak performance, particularly with respect to TN.

We next assess the computational efficiency of the proposed PGNEA in comparison with GbNEA. Table 3 reports the execution times (in minutes) of gene network enrichment analyses for Colorectal (COL), Pancreatic (PC), Gastric (GST), NSCL, and SCL cancers.

**Table 3:** Running time (in minutes) of gene network enrichment analysis across COL, PC, GST, NSCL, and SCL cancers, where the columns Network, Enrichment, and Total represent the computation times for network estimation, enrichment analysis, and the overall workflow, respectively. The runtime of GbNEA for gene network estimation was evaluated based on 200 permutations

		Network		Enrichment		Total	
		PGNEA	GbNEA	PGNEA	GbNEA	PGNEA	GbNEA
500	COL	0.3	103.9	2.9	18.1	3.1	122.0
	PC	0.2	95.5	1.9	15.3	2.2	110.7
	GST	0.2	76.3	2.5	13.7	2.6	90.0
	NSCL	0.3	103.6	2.4	30.4	2.7	134.0
	SCL	0.2	80.0	2.7	21.1	2.9	101.1
1000	COL	0.6	221.3	3.3	72.1	3.8	293.4
	PC	0.5	215.7	3.5	54.4	4.0	270.1
	GST	0.5	185.2	2.9	43.6	3.3	228.8
	NSCL	0.8	302.8	3.6	100.3	4.4	403.0
	SCL	0.5	184.3	3.0	63.0	3.5	247.3
2000	COL	1.8	739.4	4.8	231.7	6.6	971.1
	PC	1.9	754.2	4.6	203.4	6.5	957.6
	GST	1.7	674.7	4.7	193.6	6.4	868.3
	NSCL	2.4	973.4	5.3	366.3	7.8	1339.7
	SCL	1.6	629.8	4.3	196.1	5.8	825.9

As shown in Table 3, our strategy exhibits significantly enhanced computational efficiency compared with the existing GbNEA. The GbNEA requires extensive computation for permuted gene network estimations, making the process extremely time-consuming, while our method eliminates this requirement and thus achieve computation efficiency for gene network enrichment analysis.

Collectively, these results demonstrate that the proposed methodology is highly effective for gene network enrichment analysis and is a valuable tool for the identification of disease-related functional pathways.

### Uncovering viral infection pathways associated with COVID-19 severe specific molecular interplays

Coronavirus disease 2019 (COVID-19), caused by severe acute respiratory syndrome coronavirus 2 (SARS-CoV-2), is a highly contagious viral illness that has had a catastrophic impact worldwide. Similarities and dissimilarities among COVID-19 and other viral infectious diseases have gained research attention [12–14]. Numerous previous studies have suggested that well-characterized pathways from other viral infections may serve as valuable frameworks for understanding the mechanisms underlying COVID-19 pathogenesis [15, 16].

We investigated the viral infection pathways associated with the gene network in severe COVID-19 case samples. We considered other infectious diseases: viral pathways in the KEGG database are given in Table 4.

**Table 4:** Viral infection pathways in the KEGG database

Entry	Name	No. Genes
ko05171	Coronavirus disease—COVID-19	219
ko05166	Human T-cell leukemia virus 1 infection	191
ko05170	HIV-1 infection	163
ko05161	Hepatitis B	132
ko05160	Hepatitis C	112
ko05164	Influenza A	128
ko05162	Measles	117
ko05168	Herpes simplex virus 1 infection	149
ko05163	Human cytomegalovirus infection	192
ko05167	KSHV infection	169
ko05169	Epstein-Barr virus infection	172
ko05165	Human papillomavirus infection	231

The COVID-19 severe gene networks were estimated using the whole blood RNA-seq data of 1102 genotyped samples provided by the Japan COVID-19 Task Force, in which the COVID severity levels were defined as “asymptomatic (Level 1: without COVID-19 related symptoms)”, “mild (Level 2: other symptomatic patients),” “severe (Level 3: others requiring oxygen support),” and “critical (Level 4: patients in intensive care unit or requiring intubation and ventilation)” [7].

For the pathways “Coronavirus disease - COVID-19(ko05171),” we estimated COVID-19 severe and nonsevere gene networks based on the expression levels of 219 COVID-19-related genes in 303 critical and 71 asymptomatic/241 mild samples, respectively (COVID-19 networks: $G^{S}_{05171}$ and $G^{N}_{05171}$). Then, we applied our strategy to compute the statistics $t^{05171}_{j}, \quad j \in V_{05171}$, where $V_{05171}$ is the set of nodes in the COVID-19 network and the test statistic $T_{\mathrm{05171}}$. For other viral infection pathways, we estimated the gene networks based on the expression levels of severe and nonsevere samples for the genes involved in each pathway (i.e. $G^{S}_{05166}, G^{N}_{05166}, \cdots G^{S}_{05165}, G^{N}_{05165}$). Subsequently, similar analyses were performed, and the statistics $T_{05166}$, $T_{05170}$,...,$T_{05165}$ were computed. The permutation statistics $T^{*}_{k}(\omega )$ were computed by $(t^{*}_{1}(\omega ),...,t^{*}_{|V_{k}|}(\omega ))$ which is permuted $(t^{05171}_{1},\cdots ,t^{05165}_{|V_{05165}|})$ for $k=05171,...,05165$.

**Figure 3:**
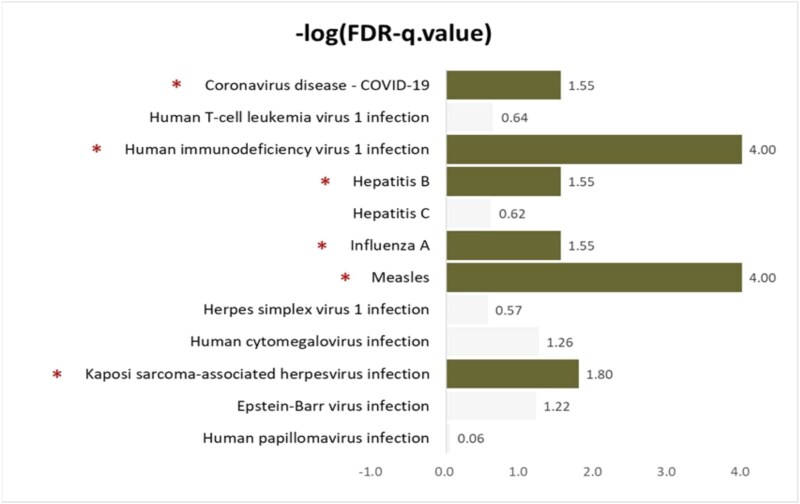
Severe COVID-19 gene network enrichment analysis results for viral infection pathways (i.e. -log(FDR-q.value), where bars with asterisks indicate significantly enriched pathways of COVID-19 sever gene network.

Next, we detected the enriched viral infection pathways based on FDR-q. values $\leq 0.05$. Figure 3 shows the $-\mathrm{log}(FDR-q.values)$ in the gene network enrichment analysis, where the green bars with asterisks indicate significantly enriched pathways (FDR-q. values $\leq 0.05$).

Our strategy identified “Coronavirus disease - COVID-19,” “Human immunodeficiency virus 1 (HIV-1) infection,” “Hepatitis B,” “Influenza A,” “Measles,” and “KSHV infection” as significantly enriched viral infection pathways in the severe COVID-19 gene networks. Interestingly, pathways related to “Human immunodeficiency virus 1 infection” and “Influenza A,” rather than “Coronavirus disease—COVID-19,” were most significantly enriched. The association between identified viral infection pathways and COVID-19 has been demonstrated in previous studies.

- Human immunodeficiency virus 1 infection
  HIV-1 primarily infects activated CD4+ T cells and myeloid cells, resulting in dysregulation of both innate and adaptive immunity, with early mucosal infection facilitating systemic viral dissemination and reservoir establishment [17]. Illanes-Álvarez *et al.* [18] revealed the similarity between HIV-1 and COVID-19; HIV-1 and COVID-19 are caused by RNA viruses of animal origin. Although the viruses differ in their modes of transmission and the symptoms they induce, they share several important similarities. These include inducing elevated levels of proinflammatory cytokines that lead to alterations in the gut microbiota and the induction of NETosis by polymorphonuclear neutrophils. In line with this, Illanes-Álvarez *et al.* [18] suggested that HIV-1 and COVID-19 have been studied in the clinical, prognostic, and therapeutic respects.
- Hepatitis B
  Hepatitis B is a serious liver infection caused by the hepatitis B virus (HBV). Infection with either SARS-CoV-2 and HBV has the potential to induce liver damage. The interaction between hepatitis B and COVID-19, mainly in the form of abnormal liver enzymes, abnormal blood parameters, and HBV reactivation, has been suggested in various previous studies [19]. Lin *et al.* [20] demonstrated that chronic HBV-infected patients in Chana showed reduced symptomatic presentation during the acute phase of COVID-19, but they experienced a greater prevalence of long-term post-COVID symptoms.
- Influenza A
  Influenza A viruses are negative-sense single-stranded RNA viruses belonging to the family Orthomyxoviridae [21]. Konala *et al.* [22] demonstrated that co-infection with influenza and COVID-19 can occur and often presents with overlapping clinical symptoms.
- Measles
  Measles is an acute viral illness transmitted via airborne droplets, marked by high infectivity and a characteristic rash, with the potential to cause serious outcomes. An evolutionary link has been proposed between coronaviruses and measles morbillivirus, based on the presence of conserved core protein structures [23]. Given the similarities between COVID-19 and other respiratory diseases, such as measles, rubella, and mumps, as well as the immunity conferred by the MMR vaccine, it was suggested that prior MMR vaccination contributed to reducing the severity of COVID-19 in children [24].
- Kaposi sarcoma-associated herpes virus infection
  Kaposi’s sarcoma, the most common cancer in untreated HIV-infected individuals, is caused by infection with KSHV, also known as human herpes virus 8 [25]. Lambarey *et al.* [26] demonstrated that HIV-infected patients with compromised immune function are susceptible to KSHV reactivation. In line with these findings, they proposed that repeated or high-level exposure to SARS-CoV-2 in unvaccinated individuals may have long-term implications for KSHV reactivation.

This suggests that the severe stages of COVID-19 may involve molecular mechanisms similar to those of other viral infections.

**Figure 4:**
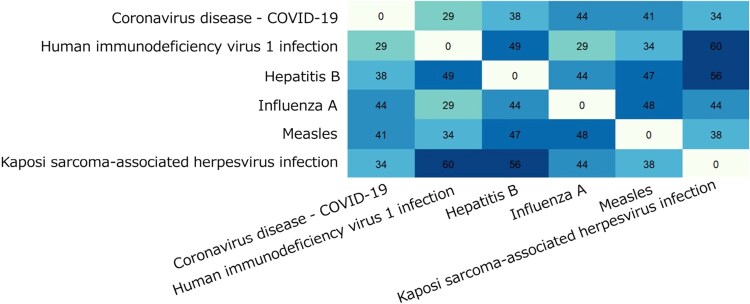
Number of overlapped genes involved in the significantly enriched viral infection pathways of the severe COVID-19 gene networks.

Figure 4 shows the shared genes involved in the significantly enriched viral infection pathways of severe COVID-19 gene networks, which comprise a total of 434 genes. As depicted in Fig. 4, 6%–14% of genes overlap within the significantly enriched pathways, and the extent of gene overlap is not as large as one might expect. The infection pathways “KSHV infection,” “HIV-1 infection,” and “Hepatitis B” exhibit molecular features similar to those observed in severe COVID-19 networks. It can be also observed that “Influenza A” exhibits the highest overlap of genes with “Coronavirus disease - COVID-19.” These findings imply that “Influenza A” and “Coronavirus disease - COVID-19” might operate via similar mechanisms, indicating that approaches previously developed for “Influenza A” could provide important clues for understanding COVID-19 severity. The full list of genes associated with the significantly enriched viral infection pathways is provided in the supplementary file.

**Figure 5:**
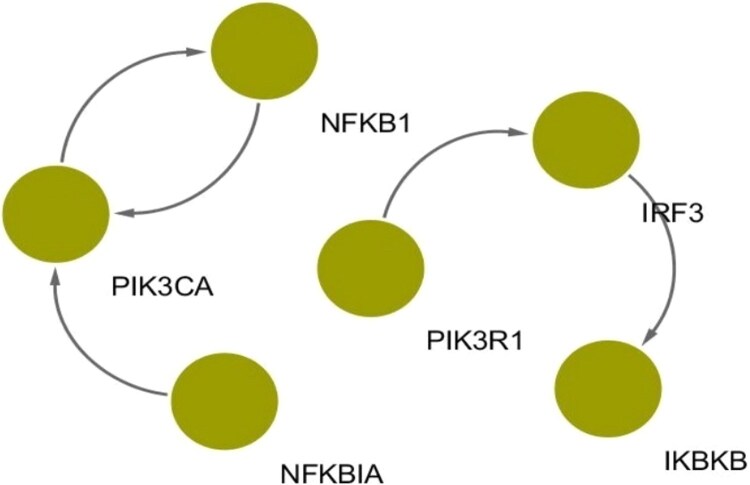
Common edges of COVID-19 severe gene networks that are significantly enriched in viral infection pathways (i.e. “Coronavirus disease - COVID-19,” “HIV-1 infection,” “Hepatitis B,” “Influenza A,” “Measles,” and “KSHV infection”).

Figure 5 shows the common molecular interplay of networks that were significantly enriched in viral infection pathways (i.e. pathways indicated by asterisks in Fig. 3). As shown in Fig. 5, interactions with PIK3 and NF-$\kappa $B families are considered characteristics of severe COVID-19 networks enriched in viral infection pathways. PIK3 and PIK3R1 are genes involved in the PI3K (Phosphoinositide 3-kinase) signaling pathway, which plays a crucial role in various cellular processes, including cell growth, survival, and metabolism [27]. NFKBIA and NFKB1 play crucial roles in the NF-B signaling pathway, which is a major regulator of inflammation and immune responses [28].

**Figure 6:**
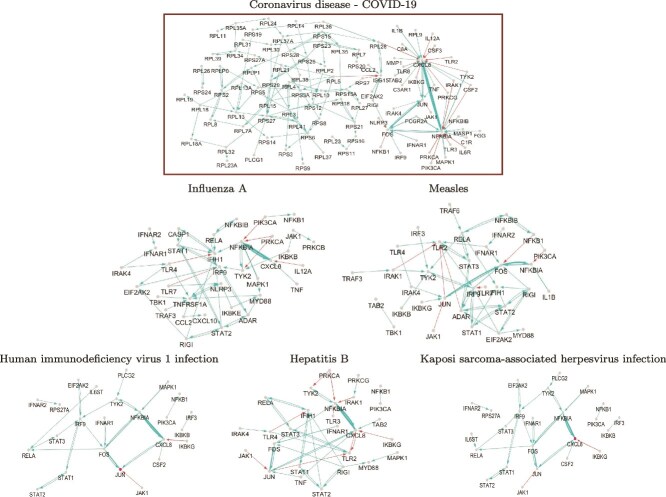
Common molecular interplays between gene networks significantly enriched in viral infection pathways with the COVID-19 pathway, where edge color indicates positive (blue) and negative (ref) effects, thickness represents the strength of the edge, and arrows ($\Box $$\rightarrow $$\triangle $) indicate that gene $\Box $ regulates gene $\triangle $.

The severe COVID-19 gene network $G^{S}_{05171}$ is shown in Fig. 6 (Top). To effectively visualize the molecular interplay, we considered only the top 5% of the absolute edge weights. The analysis highlighted CXCL8, NFKBIA, JUN, and FOS as hub genes in the COVID-19 network, among which CXCL8 and NFKBIA displayed extensive gene connectivity. The pronounced connectivity of CXCL8 and NFKBIA originates from their being regulated by a large set of genes. In other words, they serve as key target genes under the control of numerous COVID-19–related regulators. Moreover, it can be seen that CXCL8 and NFKBIA strongly regulate each other. CXCL8 and NFKBIA were well known COVID-19 markers [29–33]. In light of both our findings and prior evidence, it appears that controlling the regulators of CXCL8 and NFKBIA, either by activation or inhibition, offers a strategy to modulate these genes and consequently influence their extensive interactions. Collectively, these findings suggest that regulating the interplays involving CXCL8 and NFKBIA may offer important insights into understanding and mitigating COVID-19 severity. Furthermore, the ribosomal protein large subunit gene family has been identified as one of the main factors involved in the severe COVID-19 network.

The common molecular interplay between $G^{S}_{05161}$, $G^{S}_{05170}$, $G^{S}_{05162}$, $G^{S}_{05164}$, $G^{S}_{05167}$ and the severe COVID-19 network $G^{S}_{05171}$, as shown in the middle and bottom of Fig. 6, where only the top 25% of the absolute edge weights were included. The gene networks enriched in “Influenza A” and “Measles”-pathways show relatively higher degrees of similarity with the severe COVID-19 gene network than with the others. A strong association between CXCL8 and NFKBIA was also identified in all the common gene networks enriched in viral infection pathways and the COVID-19 gene network, except for the *measles* pathway.

- CXCL8
  Khalil *et al.* [29] suggested, based on comparative chemokine profiling of SARS-CoV-2, SARS-CoV, and MERS-CoV, that CXCL8, CXCL10, and CCL2 are key contributors to pulmonary pathogenesis in infections caused by all three coronaviruses. They also demonstrated that CXCL8 may contribute to COVID-19 pathogenesis and disease severity, highlighting its potential as a biomarker for the disease. Cambier *et al.* [30] reported that CXCL8 exists in the form of highly potent truncated proteoforms in lung transplant (LTx) patients with chronic lung allograft dysfunction and infection, as well as in individuals with COVID-19 or influenza. Hamldar *et al.* [31] also identified that dysregulation of inflammation-related genes, particularly CXCL8, may serve as a potent biomarker for managing COVID-19 infection.
- NFKBIA
  Camblor *et al.* [32] proposed that the NFKBIA variant may serve as a marker for a wider range of COVID-19 clinical manifestations, extending beyond cases associated with severe disease requiring intensive care. Simoneau *et al.* [33] identified NFKBIA, a key regulator of the NF-$\kappa $B pathway, as a universal marker of highly infected cells across all SARS-CoV-2 variants, highlighting the established link between inflammation and SARS-CoV-2 infection that underlies acute respiratory distress syndrome and severe pulmonary disease.

These results suggest that CXCL8 and NFKBIA are crucial markers of the molecular mechanisms underlying viral infections, including COVID-19. Furthermore, our results suggest that the molecular interplay between CXCL8 and NFKBIA is associated with COVID-19 severity.

**Figure 7:**
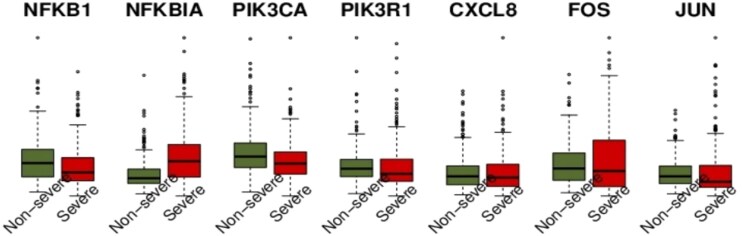
Expression levels of the identified COVID-19 severe specific markers, where read and green indicate the expression levels in nonsevere (i.e. 71 asymptomatic and 241 mild) and severe (i.e. 303 critical) samples.

Figure 7 shows the expression levels of the identified markers (i.e. PIK3 and NF-$\kappa $B family, FOX, JUN, and CXCL8) in severe and nonsevere samples.

Most of the identified markers were more highly expressed in nonsevere cases than in severe cases, with NFKBIA being a notable exception. In contrast, the markers identified exhibited increased variability in severe samples compared with nonsevere ones, with NFKBIA and FOS in particular displaying markedly higher expression variability in severe COVID-19 cases. Differential variability analysis, in addition to conventional differential expression analysis, enables the identification of disease-related genes that might otherwise be overlooked, with each approach highlighting functionally distinct sets of genes [34]. Expression variability has been suggested as an important molecular phenotype that can influence physiological traits and provide insights into disease mechanisms [35]. These observations indicate that profiling gene expression variability may offer valuable information for the development of novel diagnostic methods, therapeutic strategies, and prioritization of potential drug targets. Consistent with these findings, our results suggest that NFKBIA and FOS, which display markedly higher expression variability in severe COVID-19 cases, may serve as key markers for understanding the mechanisms underlying severe disease progression.

The upregulation of NFKBIA in severe COVID-19 samples may represent a compensatory mechanism in response to excessive activation of the NF-B pathway. NF-B plays critical roles in modulating the adaptive inflammatory response through the induction of proinflammatory genes [36], and increased NF-B activation has indeed been observed during SARS-CoV-2 infection [36]. In line with this, Simoneau *et al.* [33] reported that NFKBIA was consistently upregulated in infected cells and positively correlated with infection levels, suggesting that cells attempt to restrain NF-B activity through negative feedback. However, in severe cases, the magnitude of NF-B activation likely surpasses the capacity of this regulatory feedback, resulting in sustained inflammation and tissue damage. In contrast, nonsevere cases show upregulation of other immune-related markers, such as PIK3CA and PIK3R1, which suggest enhanced regulation of cell survival and signaling pathways that may contribute to a balanced immune response. Notably, PIK3R1, as a regulatory subunit of PI3K, plays a critical role in modulating immune cell signaling and maintaining immune homeostasis [37–39]. This pattern indicates that in milder infections, immune regulation favors coordinated signaling with a controlled inflammatory response, while in severe cases, dysregulated NF-B activity dominates, leading to a stronger reliance on inhibitory feedback through NFKBIA. Collectively, our results, in conjunction with prior studies, suggest that in severe COVID-19, dysregulated NF-B activation predominates and drives excessive inflammation, with NFKBIA upregulation reflecting an insufficient compensatory attempt to restore immune homeostasis. Conversely, in nonsevere cases, immune regulation appears to favor antiviral signaling pathways and balanced inflammatory control, highlighting the differential immune regulatory landscapes across disease severities.

Through our results and literature review, we suggest that controlling the interplay of the identified markers, especially CXCL8 and NF-$\kappa $B family, may provide crucial clues to uncover the mechanisms underlying both COVID-19 severity and viral infectious disease.

### Diverse insights into viral infection pathways within COVID-19 gene networks

To provide a broader view, we extend COVID-19 gene network enrichment analysis beyond blood to include additional tissues such as lung, bronchus, nasal cavity, and trachea, and further examine diverse cohorts spanning Asian, European, African, East Asian, South Asian, Hispanic, and Latin American populations.

We utilized single-cell RNA-seq datasets, including “A molecular single-cell lung atlas of lethal COVID-19,” “Individual Single-Cell RNA-seq PBMC Data from Guo *et al.*,” and “Large-scale single-cell analysis reveals critical immune characteristics of COVID-19 patients,” all obtained from the open data platform CZ CELLxGENE Discover (https://cellxgene.cziscience.com/). For each dataset, two phenotypes were defined (e.g. severe versus nonsevere COVID-19, or COVID-19 versus normal), and gene networks were estimated for these phenotypic groups. The analysis revealed viral infection pathways (Table 4) specifically associated with severe COVID-19 gene networks. Table 5 shows the gene network enrichment analysis results.

**Table 5:** Diverse insights into COVID-19 gene network enriched analysis results, where asterisks indicate significantly enriched pathway (i.e. FDR-q.value$\leq 0.05$) and grey values indicate non-significant pathways

Tissue	Blood	Lung	Bronchus	Nasal cavity	Trachea
Phenotype: Case	COVID-19 Severe	COVID-19	Severe		
Phenotype: Control	COVID-19 Mild/	Normal	None		
	Remission	Asian, European, Unknown,	African, East Asian,		
Cohort	Unknown	Hispanic, Latin American	European, South Asian		
Coronavirus disease—COVID-19	0.000^*^	0.000^*^	0.000^*^	0.000^*^	$\color{gray}{0.842}$
Human T-cell leukemia virus 1 infection	$\color{gray}{0.648}$	0.000^*^	0.004^*^	0.024^*^	$\color{gray}{0.300}$
HIV-1 infection	$\color{gray}{0.986}$	0.000^*^	0.004^*^	0.011^*^	$\color{gray}{0.084}$
Hepatitis B	0.048^*^	0.000^*^	0.000^*^	0.004^*^	$\color{gray}{0.561}$
Hepatitis C	$\color{gray}{0.492}$	0.000^*^	0.072	0.000^*^	$\color{gray}{0.400}$
Influenza A	0.012^*^	0.000^*^	$\color{gray}{0.077}$	0.011^*^	$\color{gray}{0.084}$
Measles	$\color{gray}{0.198}$	0.000^*^	0.000^*^	$\color{gray}{0.672}$	$\color{gray}{0.319}$
Herpes simplex virus 1 infection	$\color{gray}{0.901}$	0.000^*^	0.000^*^	0.000^*^	$\color{gray}{0.400}$
Human cytomegalovirus infection	$\color{gray}{0.492}$	0.000^*^	0.031^*^	0.004^*^	$\color{gray}{0.084}$
KSHV infection	$\color{gray}{0.498}$	0.000^*^	0.045^*^	0.011^*^	$\color{gray}{0.173}$
Epstein-Barr virus infection	$\color{gray}{0.498}$	0.000^*^	$\color{gray}{0.904}$	0.000^*^	$\color{gray}{0.561}$
Human papillomavirus infection	$\color{gray}{0.648}$	$\color{gray}{0.536}$	$\color{gray}{0.587}$	0.039^*^	$\color{gray}{0.084}$

As shown in Table 5, the severe COVID-19 gene networks in blood tissue were significantly enriched in the pathways “Coronavirus disease—COVID-19,” “Hepatitis B,” and “Influenza A,” which overlap with the pathways detected for the Japan cohort. The COVID-19 gene networks in lung tissue exhibited enrichment across almost the entire spectrum of viral infection pathways. It is also observed that no enriched pathways were detected in tracheal tissue. This finding suggests that tracheal tissue exhibits distinct molecular interactions and infection-related characteristics compared with other tissues. “Coronavirus disease—COVID-19” and “Hepatitis B” were identified as common infection pathways across most tissues, with the exception of tracheal tissue.

Collectively, the viral infection pathways associated with COVID-19 varied across different tissue types. The results highlight the need for tissue-specific monitoring and comprehensive cohort-based analyses to elucidate the mechanisms of COVID-19 from a broader perspective. We suggest through the results that insights from other viral infections may inform the understanding of mechanisms and treatment strategies for COVID-19-related lung disease.

## Discussion

We introduce a novel computational strategy called PGENA, for uncovering functional pathways associated with gene regulatory networks. Our strategy characterizes the gene network based on the coordinated actions of genes in the network, i.e. the expression levels of genes, regulatory effects, and hubness. We then measured the enrichment of a gene network based on differences in the activities of genes between phenotypes. Furthermore, our strategy assessed the significance of enrichment by permuting genes rather than phenotypes. This led to a high statistical power and computational efficiency because permuted gene network estimation was not required.

Monte Carlo simulations were employed to demonstrate the practical implementation of the proposed framework. For the cancer-related pathways in the KEGG pathways database, gene network enrichment analysis was performed. The simulation results demonstrate that the proposed PGNEA exhibits outstanding performance in identifying functional pathways enriched in a gene network. We applied our strategy to uncover the viral infection disease-related pathways enriched in the severe COVID-19 network. Using our approach, we found that several viral infection pathways—including “Coronavirus disease - COVID-19,” “HIV-1 infection,” “Hepatitis B,” “Influenza A,” “Measles,” and “KSHV infection”—were significantly enriched in gene networks associated with severe COVID-19. Additionally, our results revealed that the PIK3 and NF-B families, along with FOX, JUN, and CXCL8, serve as key markers of viral infection-related diseases. Notably, a strong association between CXCL8 and NFKBIA emerged as a recurrent signature across the enriched viral infection pathways associated with severe COVID-19 gene networks. The identified markers were validated using the existing literature. Our results suggested that targeting the identified pathways and molecular interactions may yield critical insights into the pathogenesis of severe COVID-19 and other viral infectious diseases.

The proposed PGNEA is expected to provide a powerful analytical framework for deciphering functional pathways within gene regulatory networks and to shed light on the intricate biological mechanisms that drive disease pathogenesis.

Integrating PGNEA with artificial intelligence-driven frameworks is regarded as a potential avenue for our future research. DeepInsight, developed by Sharma *et al.* [40], converts nonimage datasets (e.g. gene expression data) into structured image formats for convolutional neural networks and Vision Transformers. The method clusters similar features together while separating dissimilar ones, facilitating collective analysis of adjacent elements and potentially uncovering latent biological pathways. Our study characterizes gene networks through their individual components—regulatory influences and hub properties—and identifies enriched pathways by integrating these features. Our strategy can be enhanced by integrating DeepInsight, which maps gene network data into images to uncover biological pathways. This method utilizes the complete network information, thereby improving the efficiency and accuracy of gene network enrichment analysis.

Our approach successfully identified potential COVID-19 markers (CXCL8, NFKBIA, JUN, and FOS), which are supported by existing literature; however, experimental validation is necessary to confirm their functional roles. As our study is computational, such validation could not be conducted in our laboratory, representing a limitation of the current work. Nevertheless, we expect that these findings will offer valuable insights into COVID-19 and can be further validated and expanded through wet-lab studies to uncover functional markers.

**Key Points**

- A novel computational strategy for gene network enrichment analysis called PGNEA is introduced.
- The proposed strategy is applied to uncover viral infection pathways associated the sever COVID-19 gene network.
- A strong association between CXCL8 and NFKBIA was consistently observed in enriched viral infection pathways associated with severe COVID-19 gene networks.
- PGNEA may serve as a valuable tool for the identification of disease-related functional pathways.

## Supplementary Material

Supplimentary_file_of_PGNEA_bbaf647

## Data Availability

The code and toy data to implement PGNEA are available in Figshare. https://doi.org/10.6084/m9.figshare.30183259.
